# Detection and localization of calcium oxalate in kidney using synchrotron deep ultraviolet fluorescence microscopy

**DOI:** 10.1107/S1600577521011371

**Published:** 2022-01-01

**Authors:** Emmanuel Estève, David Buob, Frédéric Jamme, Chantal Jouanneau, Slavka Kascakova, Jean-Philippe Haymann, Emmanuel Letavernier, Louise Galmiche, Pierre Ronco, Michel Daudon, Dominique Bazin, Matthieu Réfrégiers

**Affiliations:** a Sorbonne Université, UPMC Paris 06, Institut National de la Santé et de la Recherche Médicale, Unité Mixte de Recherche S 1155, F-75020 Paris, France; b AP-HP, Hôpital Tenon, Department of Nephrology and Dialysis, Paris, France; c AP-HP, Hôpital Tenon, Department of Pathology, Paris, France; d Synchrotron SOLEIL, DISCO Beamline, L’Orme des Merisiers, Saint-Aubin, 91192 Gif sur Yvette, France; e Département Hospitalo – Universitaire Hepatinov, Université Paris-Saclay, UMRS 1193, INSERM, Villejuif, France; f AP-HP, Hôpital Tenon, Department of Physiology, Paris, France; g Pathology Department, Necker-Enfants Malades Hospital, Public Assistance-Hospitals of Paris, Université Paris, 75015 Paris, France; h Laboratoire de Physique des Solides, CNRS UMR8502, Université Paris Saclay, Orsay, France; i CNRS-LCMCP-Sorbonne Universités-UPMC Univ Paris 6, Collège de France, Paris, France; j CNRS, Institut de Chimie Physique, Université Paris-Saclay, Orsay, France; k Centre de Biophysique Moléculaire, CNRS UPR4301, Rue Charles Sadron, Orléans, France

**Keywords:** oxalate, oxalosis, deep ultraviolet microscopy, synchrotron diagnosis, kidney biopsies, synchrotron diagnosis

## Abstract

Renal oxalosis is a rare cause of renal failure whose diagnosis can be challenging. Synchrotron deep ultraviolet (UV) fluorescence was assayed to improve oxalosis detection on kidney biopsies spatial resolution and sensitivity compared with the Fourier transform infrared microspectroscopy gold standard. The fluorescence spectrum of synthetic mono-, di- and tri-hydrated calcium oxalate was investigated using a microspectrometer coupled to the synchrotron UV beamline DISCO, Synchrotron SOLEIL, France. The obtained spectra were used to detect oxalocalcic crystals in a case control study of 42 human kidney biopsies including 19 renal oxalosis due to primary (PHO, *n* = 11) and secondary hyperoxaluria (SHO, *n* = 8), seven samples from PHO patients who received combined kidney and liver transplants, and 16 controls. For all oxalocalcic hydrates samples, a fluorescence signal is detected at 420 nm. These spectra were used to identify standard oxalocalcic crystals in patients with PHO or SHO. They also revealed micrometric crystallites as well as non-aggregated oxalate accumulation in tubular cells. A nine-points histological score was established for the diagnosis of renal oxalosis with 100% specificity (76–100) and a 73% sensitivity (43–90). Oxalate tubular accumulation and higher histological score were correlated to lower estimated glomerular filtration rate and higher urinary oxalate over creatinine ratio.

## Abstract 2

Synchrotron deep ultraviolet imaging highlighting pathological calcium oxalate detection in kidney is presented.

## 1. Introduction

Various conditions may induce high oxalate excretion by the kidneys and subsequent calcium oxalate crystal (CaOxC) deposition in kidney tubules and interstitium, referred to as renal oxalosis (Yuan *et al.*, 1992 ▸; Getting *et al.*, 2013 ▸). Oxalate is a tiny molecule absorbed in the small and large intestine. Renal oxalosis can be due to primary hyperoxaluria (PHO) which is a rare genetic disease that causes recurrent nephroli­thia­sis, nephrocalcinosis, systemic oxalosis, and end-stage renal disease (ESRD) (Cochat & Rumsby, 2013 ▸). Any gut pathology leading to fat malabsorption such as inflammatory bowel disease (Hueppelshaeuser *et al.*, 2012 ▸), bariatric surgery (Nasr *et al.*, 2008 ▸) or chronic pancreatitis (Cartery *et al.*, 2011 ▸) can lead to secondary hyperoxaluria (SHO), renal oxalosis, and ESRD (Robijn *et al.*, 2011 ▸).

Clinical course of the disease, oxalate dosage in blood and urine, crystalluria, and genetic findings can lead to accurate diagnosis and treatment (Lieske *et al.*, 2005 ▸; Daudon *et al.*, 2008 ▸; Zhao *et al.*, 2016 ▸). However, diagnosis is often missed or delayed by several years and can be challenging in end-stage renal disease patients (Daudon *et al.*, 2008 ▸). In 2014, among the 405 patients enrolled in the Rare Kidney Stone Consortium Primary Hyperoxaluria Registry, 112 (27%) patients had reached ESRD at the time or before diagnosis (Zhao *et al.*, 2016 ▸). In a previous report of the registry, oxalosis was even diagnosed after recurrence on the transplant in seven out of 95 patients (Lieske *et al.*, 2005 ▸).

Besides clinical and biological features, identification of numerous CaOxC in the kidney is the central hallmark of renal oxalosis diagnosis. CaOx are not stained by usual techniques (Herlitz *et al.*, 2012 ▸) and can easily be missed if polarized microscopy (PM) is not or is incorrectly performed. Although pathological characteristics can help to differentiate among different crystals found in renal biopsies (Herlitz *et al.*, 2012 ▸; Nasr *et al.*, 2010 ▸), definitive chemical composition can only be determined by physico-chemistry approaches (Estepa-Maurice *et al.*, 1996 ▸; Bollée *et al.*, 2012 ▸; Bazin *et al.*, 2012 ▸). Among them, Fourier transform infrared microspectroscopy (FTIRM) is the most commonly used. It is highly effective but its resolution and therefore sensitivity is diffraction limited to 3–6 µm (Dessombz *et al.*, 2011 ▸).

We propose a new, high-sensitive technique based on synchrotron deep UV (DUV) microspectroscopy, which opens new perspectives for the diagnosis of oxalate-mediated kidney diseases and the understanding of their physiopathology.

## 2. Materials and methods

### 2.1. Patients

Patients’ clinical, biological and main histological data are reported in Tables S1 and S2 of the supporting information.

Clinical and biological information were retrospectively retrieved from clinical files. PHO diagnosis was either confirmed by genetic analysis and/or decreased alanine:glyoxylate amino­transferase activity on liver biopsy. SHO diagnosis was proposed when renal oxalosis was diagnosed in the context of either ethyl­ene glycol intoxication or digestive malabsorption and in the absence of familiar evidence orienting towards PHO. Delay between biopsy and combined liver/kidney transplantation is specified in Table S1. As stipulated in Table S1, kidney biopsies used as control came from our standard clinical activity and included various profiles of normal and pathological native or transplanted kidneys.

The estimated glomerular filtration rate (eGFR) was estimated using the closest available serum creatinine (within one month) to the biopsy, and the modification diet in renal disease formula for adults and the Schwartz formula for children (Levey *et al.*, 1999 ▸; Schwartz *et al.*, 2009 ▸).

An ethical committee was not required for this observational study according to Helsinki law and the French Institutional Committee. Patients were informed by the time the biopsy was sampled that kidney tissue could be used for scientific purpose and consented to this study.

### 2.2. Biopsies preparation

Renal biopsies were fixed in a formalin acetic alcohol solution (1% acetic acid/ethanol 70°/10% formalin) for 3 h. Dehydration was performed in a carousel (Microm France STP MM 120) before paraffin (Leica/ParaplastSurgipath) inclusion. Three-micrometre-thick slices were spread on low-e microscope slides (Kevley Technologies, Tienta Sciences, USA). A first drying was operated at 56°C for two hours followed by two successives 15 min baths in xylene (Carlo Erba) to remove paraffin. Synthetic CaOx powders [calcium oxalate monohydrate (COM) or whewellite, dihydrate (COD) or weddellite and thrihydrate (COT) or caoxite synthetized in-house] were deposited on quartz coverslips (ESCO, USA).

### 2.3. Beamline optical setup

DUV fluorescence microspectroscopy was performed on a UV microspectrometer coupled to the monochromated synchrotron beam at the SOLEIL DISCO beamline (Giuliani *et al.*, 2009 ▸). After excitation at 275 nm, each spectrum was collected during 2 s in the 300–500 nm spectral range. The rastering step between two spectra was 2 µm and typical spectral maps were in the 50 µm × 50 µm range.

The DUV wide-field imaging system has been thoroughly described previously (Jamme *et al.*, 2013 ▸). Fluorescence images were recorded with 5 s exposure. The whole system is controlled by *μManager* (Edelstein *et al.*, 2010 ▸).

Polarized light images were acquired on a BX45 Olympus microscope (Olympus, France) equipped with a polarizing system and a compensatory lens.

FTIRM was performed on a Spotlight 400 FTIR imaging system (Perkin-Elmer). Spectra were collected in reflection mode between 4000 and 700 cm^−1^, each spectrum being acquired after 64 accumulations at 8 cm^−1^ resolution. Images used for oxalosis diagnosis were acquired with a 6.25 µm spatial resolution, the maximal resolution allowed by refraction within this wavelength regimen (Petit *et al.*, 2018 ▸).

All image calculations described in the manuscript were performed using *FIJI* (Schindelin *et al.*, 2012 ▸).

All wide-field DUV fluorescence (WFUV) images were blindly analysed by two independent readers. Biopsies were considered positive for macrocrystals if one crystal >10 µm was seen. Microcrystal presence was estimated on a semiquantitative four-point scale [0: no crystal; 1: scarce crystals (<3 per map); 2: few crystals (3–10 per map); 3: numerous microcrystals (>10 per map)].

### 2.4. Statistical analysis

All statistics analysis was performed using *GraphPad Prism* version 8.00 for Windows (GraphPad Software, La Jolla, California, USA).

Sensitivity and specificity are presented with a 95% confidence interval. The statistical significance of contingency tables was evaluated using two-sided Fisher’s exact test. As the percentage of positive tubular sections does not seem to follow a normal distribution, statistical significance was evaluated using the Kruskal-Wallis test followed by comparison of the mean rank using Dunn’s multiple comparisons test. To avoid pseudoreplication and artificial statistical power, tubular fluorescence ratio were compared using nested t-test. Medians are presented with 25% and 75% percentile.

Correlations between the oxaluria, eGFR and WFUV results were analysed using linear regression. 95% confidence interval of the slope is represented by dashed lines.

Uric acid and calcium oxalate mono- and di-hydrate kidney stone fluorescence spectra were treated using lowess spline fitting using 20 points in the smoothing window.

## 3. Results and discussion

### 3.1. Synchrotron UV fluorescence spectra of synthetic CaOx can be retrieved from kidney biopsy

**Figure 1:**
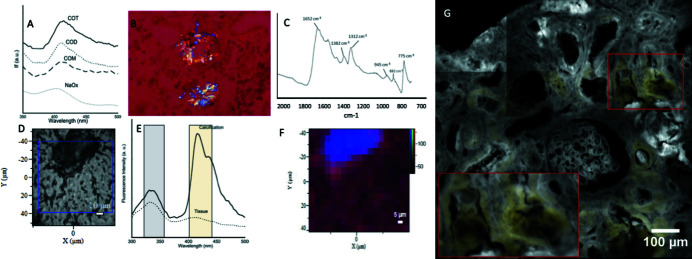
UV fluorescence can be used to detect calcium oxalate. (A) Synthetic calcium oxalate monohydrate (COM), dihydrate (COD) and thrihydrate (COT) fluorescence spectra after deep UV excitation (275 nm). (B) Typical refringent tubular crystal under polarized light microscopy suggestive of calcium oxalate deposit (polarized light with quarter-wave blade, real colours). (C) FTIR spectra of the crystals confirm that COM is the main chemical phase involved. (D) Phase contrast microscopy is used to spot these crystals under the UV spectral microscope beam. (E) Under UV excitation, a 420 nm emission peak is retrieved in the calcification. This emission peak is absent in the surrounding tissue. Therefore, if a 327–353 nm filter (grey box) allows background tissue imaging (aromatic amino-acid autofluorescence) a 412–438 nm filter (yellow box) could allow CaOx imaging. (F) The spatial distribution of this peak matches phase contrast crystal mapping (RGB reconstituted, 420 nm peak intensity in blue and 330 nm peak in red). (G) As predicted by the spectra this fluorescence peak can be explored using an inverted fluorescence microscope. The 327–353 nm channel (aromatic amino-acid fluorescence in grey) allows recognition of the kidney architecture. The 412–428 nm signal is only higher than the 327–353 nm signal within the CaOx crystals. Subtracting the 327–353 nm map from the 412–438 nm (yellow) allows generation of a potential CaOx signal.

Synchrotron UV fluorescence emission spectra collected from synthetic samples showed that CaOx had a specific fingerprint centred at 420 nm, separated from aromatic acids autofluorescence [Fig. 1 ▸(A)]. It was, however, not possible to discriminate the CaOx hydration level. To confirm that UV fluorescence microspectroscopy could be used on standard formalin-fixed paraffin-embedded human kidney biopsy, we challenged it with a single, massive FTIRM-confirmed CaOx crystallite [Figs. 1 ▸(B), 1 ▸(C) and 1 ▸(D)]. FTIR [Fig. 1 ▸(C)] shows a 1591–1623 cm^−1^ massif typical of oxalate vibrations (Petit *et al.*, 2018 ▸) and several COO vibrations between 500 and 1000 cm^−1^. After recording a set of UV emission spectra at 275 nm excitation, we found the 420 nm CaOx fluorescence peak identified in synthetic compound [Fig. 1 ▸(E)] and the spatial representation of its intensity matched the crystal [Fig. 1 ▸(F)]. UV fluorescence gives a signature of the S1 → S0 transition from molecules that present at the same time a conjugated pi system and a chemical group able to donate one electron. Oxalates per se do match these prerequisites; therefore the 420 nm emission is likely independent of its involvement in a crystallite organization.

To explore larger areas of the same biopsy specimen, we performed wide-field synchrotron DUV fluorescence. Signal was recorded after 275 nm excitation using two bandpass filters: 427–438 nm (attributed to oxalate) and 327–353 nm (attributed to aromatic amino acid) [Fig. 1 ▸(E)]. As predicted by the tissue spectra [Fig. 1 ▸(E)], in non-pathologic conditions, the signal was stronger in the 327–353 nm than in the 427–438 nm channel. To isolate the areas where this ratio was inverted, we calculated the difference in intensity between the 327–353 nm and 427–438 nm signals for each pixel of the fluorescence map. The result of this subtraction is represented in yellow above the 327–353 nm amino-acid related background signal [Fig. 1 ▸(G)]. This signal in the test biopsy matches oxalate accumulation and underlines several large CaOx deposits surrounding damaged tubules. The large red square presents a close-up of an area identified by the smaller red square where two tubular sections filled by a crystal that led to their destruction and protrude in the tubular lumen can be seen.

### 3.2. Wide-field synchrotron DUV fluorescence for oxalate screening in kidney biopsies and surgical specimens

To assess the WFUV potential in detecting oxalate in kidney tissue, we performed a case control study acquiring large fluorescence maps of 38 kidney biopsies and four nephrectomy specimens from 39 patients (Tables S1 and S2). Renal oxalosis biopsies were either retrieved from the bio­repository of samples referred to one of us (MD) for determination of the chemical phase in abnormal tissue deposits (*n* = 11) or after systematic search for ‘oxalosis’ in the kidney biopsy registries of Tenon and Necker hospitals, both in Paris, France (*n* = 9 and 6, respectively). Oxalosis was due either to PHO on native kidney (*n* = 7) or on kidney transplant (in the absence of combined liver transplantation) (*n* = 4) or to secondary hyperoxaluria (SHO) (*n* = 8). Seven biopsies were obtained from renal allografts of five patients with PHO treated by kidney and liver transplantation (KLTPHO). Controls were from the FTIRM-characterized biopsy bank (MD, *n* = 4) and from the Tenon Pathology department (*n* = 12). The control group included non-oxalosis crystals containing biopsies (*n* = 5), tubulointerstitial nephritis (*n* = 5) and glomerular diseases (*n* = 3) and preimplantation kidney transplant biopsies considered as normal (*n* = 3). Concise clinical, biological and histological details are presented in Tables S1 and S2.

**Figure 2:**
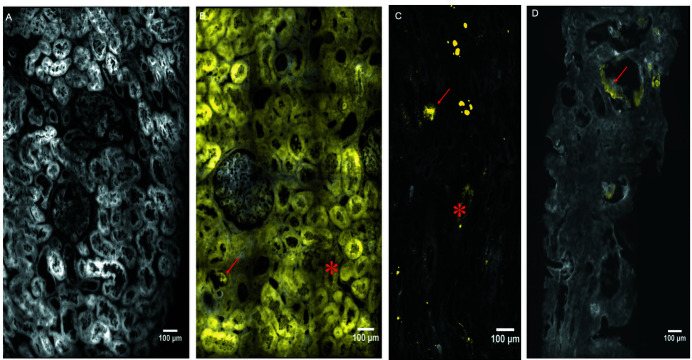
Synchrotron WFDUV mapping of typical kidney biopsies. The signal (yellow) was obtained after subtraction of the 412–438 nm channel and 327–353 nm channel fluorescence map. The background signal (grey) was obtained using the 327–353 nm channel (aromatic amino-acid fluorescence). (A) Normal kidney. (B) Primary hyperoxaluria patients present large CaOx crystals (arrow), small punctiform structures compatible with microcrystals (asterisks) and a diffuse signal in the cytoplasm of tubular cells. (C) Secondary hyperoxaluria have either large deposits (arrow) and punctiform signals (asterisks) without clear tubular diffuse distribution or (D) only large deposits (arrow).

In normal tissue, the 412–438 nm signal was inferior to the 327–353 nm signal. Therefore, in typical negative controls, the 427–438 to 327–353 nm subtraction was negative in every pixel [Fig. 2 ▸(A)]. Three patterns were associated with renal oxalosis: a large, strong signal suggestive of typical CaOx aggregates, a strong punctiform signal suggestive of micrometric crystals, and a more diffuse signal within the cytoplasm of tubular cells [Figs. 2 ▸(B), 2(C), 2(D)]. In primary hyperoxaluria patients [Fig. 2 ▸(B)], the signal is observed in large formations within flattened epithelia tubules suggestive of large CaOx crystals (arrow), in small punctiform structures compatible with microcrystals (asterisk), and is diffuse in the cytoplasm of tubular cells. In secondary hyperoxaluria [Figs. 2 ▸(C) and 2(D)], large deposits (arrow) and punctiform signals (asterisks) are also observed but tubular diffuse enhancement is inconstant. Two blinded readers analysed these patterns on all images (see the *Materials and methods* section[Sec sec2] for experimental details).

Four non-oxalosis biopsies were difficult to interpret (patients 36–39). In these biopsies, the 427–438 nm signal was stronger all over the underlying histological architecture including the glomerular tuft. These biopsies were sampled from the two patients with 2,8-di­hydroxy­adenine crystal nephropathy [Figs. S1(A) and S1(B) of the supporting information], one patient with suspected autosomal dominant tubulointerstitial kidney disease (familial records of kidney disease, juvenile gout with hyperuricemia, chronic tubulointerstitial nephritis) [Fig. S1 ▸(C)], and one patient with crystal-storing histiocytosis [Fig. S1 ▸(D)]. Therefore, these biopsies were not included in the statistical analysis.

### 3.3. Synchrotron DUV can identify macro and micro deposits in renal oxalosis patients

**Figure 3:**
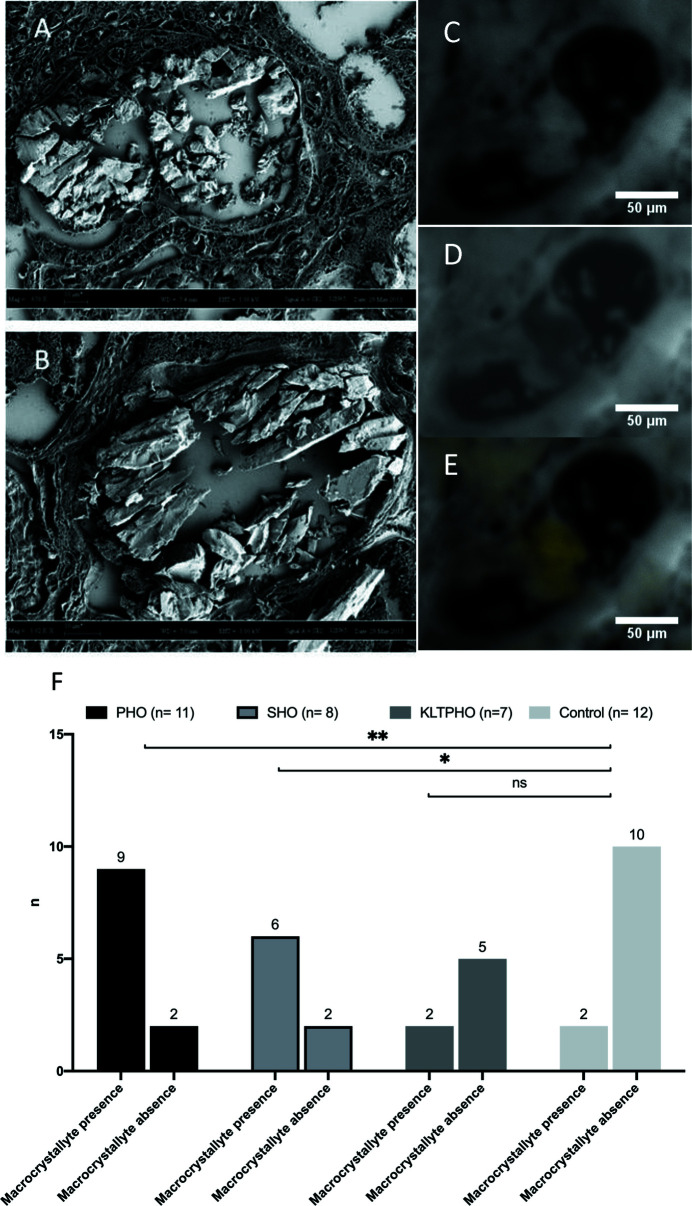
Synchrotron WFUV allows detection of CaOx macrocrystallites in kidney biopsies. (A, B) Scanning electron microscope observations of typical CaOx macrocrystallites in the tubule lumens. Fluorescence is bright in the 412–438 nm channel (C) and shallow in the 327–353 nm channel (D) which results in a yellow subtract signal (E) evocative of CaOx composition. (F) Macrocrystal presence is significantly more frequent in PHO and SHO patients than in control biopsies, while it is not frequent in KLTPHO patients. ** *p* < 0.01; * *p* < 0.05; ns: not significant.

Large deposits suggestive of macrocrystallite (diameter: 10–75 µm) were found in 19 biopsies [15 renal oxalosis (PHO + SHO), 2 KLTPHO, and 2 controls: patients #27 (isolated hematuria, normal kidney) and #34 (acute cellular transplant rejection, FTIRM confirmed CaOx crystals)]. They were almost always observed in tubular lumen or in tubular cells. We performed scanning electron microscope (SEM) analysis to confirm that the large positive formations matched big stone-like formations located in severely injured tubular sections [Figs. 3 ▸(A), 3(B)]. Interestingly in the centre of these aggregates, the signal in the aromatic amino acid channel was very low suggesting that the core of the aggregate contained few proteins. The aromatic signal was, however, high in the periphery of the aggregate suggesting potential proteic corona [Figs. 3 ▸(C)–3(E)].

Macrocrystallites were statistically more recurrent in the case of primary (*p* = 0.003) or secondary hyperoxaluria (*p* = 0.02) than in controls. Macrocrystallites were noted in 9/11 (82%) PHO biopsies, 6/8 (75%) SHO biopsies, 2/7 (29%) KLTPHO biopsies and 2/12 (17%) control biopsies [Fig. 3 ▸(F)] including patient #34.

**Figure 4:**
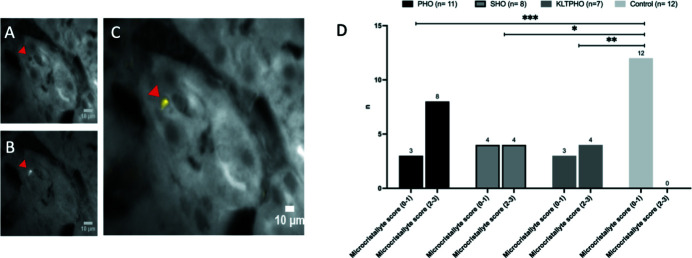
Synchrotron WFUV allows detection of CaOx microcrystallites in kidney biopsies. The bright spot in the 412–438 nm channel (A), indicated by the arrowhead, corresponds to a focal shadow in the 327–353 nm channel (B) resulting in a strong yellow spot after signal subtraction (C). (D) Frequency of the semi-quantitative microcrystal score [0: no crystal; 1: scarce crystals (<3 per map); 2: few crystals (3–10 per map); 3: numerous microcrystals (>10 per map)] in PHO, SHO and KLTPHO and control biopsies. *** *p* < 0.001; ** *p* < 0.01; * *p* < 0.1.

Small punctiform signals suggestive of micrometric crystallites undetected by FTIRM were identified in 26 of the 38 analysed biopsies [16 renal oxalosis, 5 KLTPHO, and 5 controls (patients #24, #25, #26, #27 and #35; see Table S1 and S2 for clinical information)]. They were frequently found in the tubular lumen (24/26) and in the cytoplasm of tubular cells (21/26), but they could also be observed in the interstitium (18/26), small vessels (2/26) and glomeruli (5/26). On higher magnification, some of these microdeposits were shown to be intracellular [Figs. 4 ▸(A)–4(C)]. As seen in larger aggregates, the punctiform reinforcement of the fluorescence in the oxalate channel corresponds to a local shadow in the aromatic amino acid channel which suggests that local oxalate saturation pushes away normal intracellular protein content [Figs. 4 ▸(B)–4(C), arrowhead].

Micrometric crystallite density was quantified using a semi-quantitative score on a 0 to 3 scale. Scarce microcrystallites were often spotted in control biopsies [5/7 (71%)] but no control biopsy was scored above 1. A 2 or 3 microcrystallite density score was granted in 8/11 (73%) PHO, 4/8 (50%) SHO and 4/7 (57%) KLTPHO biopsies [Fig. 4 ▸(D)]. A semi-quantitative microcrystal score above 2 is significatively more frequent in PHO, SHO and KLTPHO and never granted on control biopsies.

### 3.4. Synchrotron DUV fluorescence can identify oxalate accumulation in tubular cells

**Figure 5:**
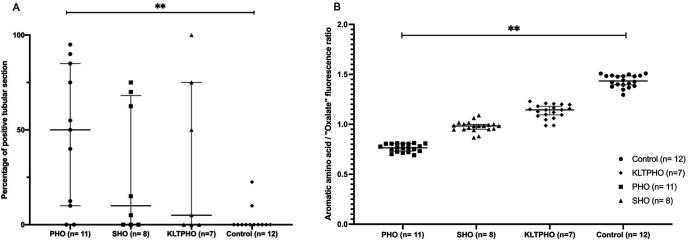
Diffuse intracytoplasmic tubular signal is associated with renal oxalosis. (A) Percentage of positive tubular section. Fluorescence intensity ratio between the ‘oxalate’ and aromatic amino acid channel was measured in 20 positive tubular cell cytoplasm regions for each biopsy. (B) Median with 25–75 interquartile range. ** *p* < 0.01.

Although diffuse intracellular signal was not expected, a tubular signal appeared in 9/11 PHO [median 50% of positive tubular section (IQ25-75 10–85)], 5/8 SHO [median of positive tubular section 10% (IQ25-75: 0–68)], and 4/7 KLTPHO [median of positive tubular section 5% (IQ25-75: 0–75)] biopsies. The percentage of involved tubules ranged from 5 to 100% [Fig. 5 ▸(A)]. A tubular signal was only observed in 2 of the 12 ‘control’ biopsies (patients #33 and #34 with transplanted kidneys and a percentage of positive tubules of 10 and 22%, respectively). Therefore, the percentage of positive tubules was significantly higher in PHO patients than in control biopsies.

To better quantify this signal, 20 punctiform regions of interest (ROIs) were randomly selected within tubular cytoplasm for each biopsy. The fluorescence intensity ratio between the 327–353 nm channel corresponding to tissue background and the 427–438 nm channel related to oxalate was measured for each of these spots [Fig. 5 ▸(B)]. As expected in control biopsies, this ratio was above 1 [median 1.21 (IQ25-75 0.99–1.77)]. In PHO mediated renal oxalosis, this ratio significantly decreased to 0.72 (IQ25-75 0.62–0.95) (*p* < 0.01). This ratio trended towards higher value in SHO, 0.88 (IQ25-75 0.78–1.07), and KTLPHO, 1.08 (IQ25-75 0.80–1.43), than in PHO, although not reaching control levels suggesting possible correlation with the severity of the disease (ns). This ratio was even lower in patients #36–39 (*i.e.* the patients that we had to withdraw from further analysis due to odd signal with a median at 0.42 (IQ25-75: 0.30–0.58) (*p* < 0.1) confirming that the signal seen in these samples is different (Fig. S3 of the supporting information).

### 3.5. Wide-field synchrotron DUV histological score for oxalosis diagnosis in kidney biopsies

We built up a nine-points score that combines macrocrystallite presence, microcrystallite staging, and tubular fluorescence positivity in a single element.

**Figure 6:**
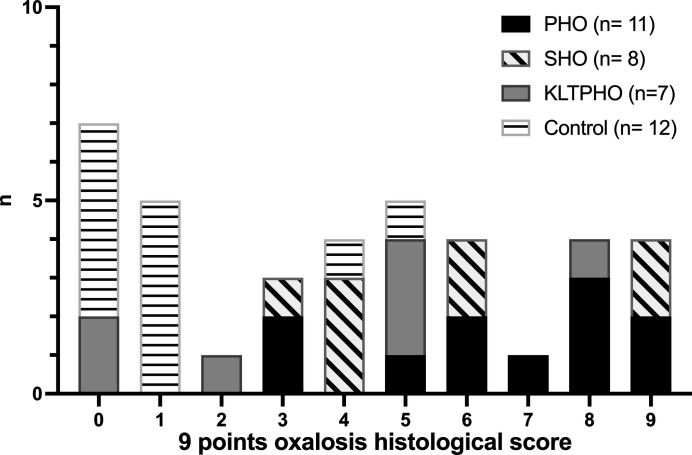
Nine-points oxalosis histological score allowed accurate renal oxalosis diagnosis. Frequency distribution of the patients according to various oxalosis subgroup and their histological grading.

Macrocrystallite presence was granted 3 points. Microcrystallite score ranged from 0 to 3 points staging used as specified in the dedicated session. A tubular fluorescence score was established according to the percentage of positive tubular section (0%: 0; <10%: 1; 10–50%: 2; >50%: 3). This composite score frequency distribution is reported in Fig. 6 ▸. Most oxalosis patients were given high scores: median 7 points (IQ25-75 5–8, *p* < 0.001), 5 points (IQ25-75 4–8.25, *p* < 0.05) and 5 points (IQ25-75 0–5, ns) for PHO, SHO and KLTPHO patients, respectively, compared with a median 1 point (IQ25-75 0–1) for controls. Only patients with PHO or SHO achieved maximum scoring.

A 6 points score or above allows diagnosis of PHO with 100% specificity (76–100) and a 73% sensitivity (43–90) (Table 1 ▸). The full contingency table is given as Table S3 of the supporting information.

### 3.6. Oxalate detected by wide-field synchrotron DUV is associated with lower glomerular filtration rate and higher urinary oxalate/creatinine ratio in renal oxalosis patients

Estimated Glomerular Filtration Rate (eGFR) was only available for 27 of the included patients (PHO, *n* = 5; SHO, *n* = 6; KLTPHO, *n* = 7; control, *n* = 9) limiting our ability to correlate our histological observation with renal function.

eGFR seemed comparable between most groups but, when pooled together, as expected, oxalosis patients had lower eGFR than controls (*p* < 0.05) (Fig. S2 of the supporting information). Five patients in the PHO groups had reached end-stage renal disease by the time of kidney sampling, granting them a 0 mL min^−1^ eGFR exacerbating this difference but not impacting subsequent analysis.

**Figure 7:**
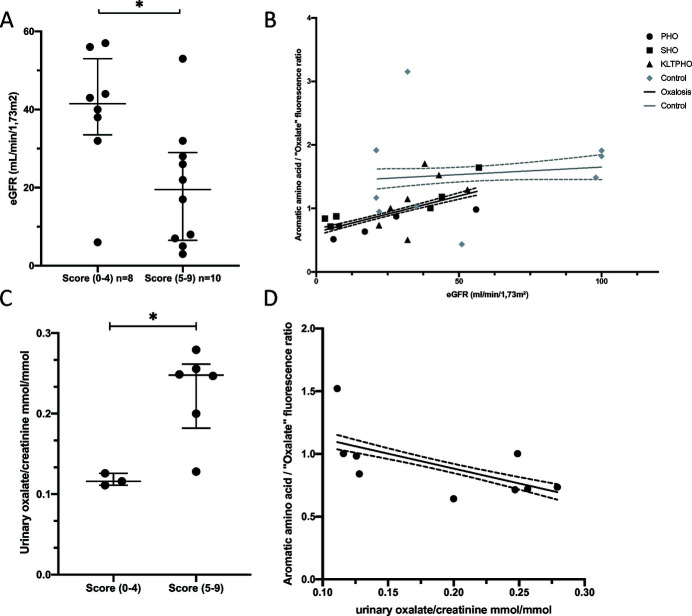
eGFR and oxaluria correlation with WFUV oxalate imaging. (A) In oxalosis patients, a higher nine-point oxalosis histological score is associated with lower eGFR. (B) In oxalosis patients, a lower ‘aromatic amino acid’/ ‘oxalate’ tubular fluorescence ratio is associated with lower eGFR (linear regression: black line; dashed line IC95, *p* < 0.0001) whereas in control patients both variable are independents (linear regression: grey line; dashed line IC95). (C) In oxalosis patients, higher nine-points oxalosis histological score is associated with higher urinary oxalate/creatinine ratio. (D) High urinary oxalate/creatinine ratio is associated with lower ‘aromatic amino acid’/ ‘oxalate’ tubular fluorescence ratio (linear regression: black line; dashed line IC95, *p* < 0.0001). Median with 25–75 interquartile range. * *p* < 0.05.

Patients presenting the lowest nine-points histological score, *i.e.* (0–4) (PHO, *n* = 2; SHO, *n* = 3; KLTPHO, *n* = 3), had a significantly higher eGFR [median 41.5 ml/min/1.73 m^2^ IQ25-75 (33.5–53)] compared with patients with high scores (5–9) (PHO, *n* = 3; SHO, *n* = 3; KLTPHO, *n* = 4) [median 19.5 mL/min/1.73 m^2^ (IQ25-75 6.5–29), *p* < 0.05] [Fig. 7 ▸(A)]. A higher cut-off (0–5) versus (6–9) led to even more significant results but almost all KTLPHO patients were given a low score. Since only two control patients have a 4 or above nine-points oxalosis histological score, statistical significance is not warranted but eGFR was not lower for this subpopulation [Fig. S2(B)].

Given the low number of patients and scarce data, we could not perform multivariate analysis and rule out confounding bias.

We could only retrieve urinary oxalate/creatinine ratios from nine oxalosis patients. Patients with high nine-points histological score (5–9) had higher urinary oxalate creatinine ratio [median 0.25 mmol/mmol IQ25-75 (0.18–0.26)] than patients with lower score [median 0.12 mmol/mmol IQ25-75 (0.11–0.13), *p* < 0.05] [Fig. 7 ▸(C)]. The tubular fluorescence intensity ratio was inversely correlated with the urinary oxalate creatinine ratio (*p* < 0.0001) [Fig. 7 ▸(D)].

## 4. Discussion

In this study, we show that oxalate fluorescence emission around 420 nm after 275 nm synchrotron DUV excitation can be used to identify oxalate in kidney biopsies. This method allows detection of the expected macrocrystals but also numerous tubular and extratubular microcrystals in renal oxalosis patients. Furthermore, we identified for the first-time oxalate accumulation in the cytoplasm of tubular cells. These findings could be combined in a nine-points histological score.

Standard microscopy using chemical or immunological staining of kidney biopsy has led to a better understanding and classification of kidney diseases (D’Agati & Mengel, 2013 ▸). However, standard staining techniques can only provide structural information. When a crystal is found in a kidney biopsy, although birefringence after polarized light observation, shape of the crystal, and its affinity to various stains can help diagnosis, it is not specific enough to formally establish its chemical phase (Falk *et al.*, 2013 ▸).

Deep UV fluorescence spatial resolution is submicrometric. It gives less chemical information than FTIR but is more sensitive and better spatially resolved. It does not require specific sample preparation (standard glass slide can be used if a quartz coverslip is mounted on it). Large areas of the biopsy can be investigated. Unlike mass spectrometry, it is not destructive and can be conveniently coupled with other standard microscopic techniques (Bazin *et al.*, 2016 ▸; Petit *et al.*, 2010 ▸; Zubkovs *et al.*, 2014 ▸). However, UV fluorescence data analysis of complex biological samples can be challenging. Many molecules can be excited by UV and emit fluorescence in the window of interest (Jamme *et al.*, 2010 ▸). So far, there is no exhaustive UV fluorescence catalogue. Therefore, signal specificity is always a concern.

Four biopsies were excluded before analysis due to diffuse positive signal suggestive of non-oxalate related fluorescence (patients #36 to #39; see Fig. S2 for details). This diffuse signal was histologically very different. It covered all tissue regardless of underlying kidney architecture suggesting diffuse impregnation. The fluorescence ratio was significantly lower than in any other biopsy suggesting excitation of a different fluoro­phore. Biopsies were not processed the same day. They were sent to Tenon hospital from various institutions – therefore pre-analytical flaw is not likely. Interestingly, all these patients shared urate metabolism abnormalities, and uric acid fluorescence spectra seem to potentially overlap oxalate fluorescence spectra (see Fig. S3 for further description of these biopsies) which calls for a dedicated study. Identification of these biopsies was in fact quite easy to the expert eye although it raises concern regarding signal specificity.

The large tubular deposits evocative of CaOx macrocrystals detected by subtracting the 327–353 nm channel signal from the 412–438 nm ones correlate with usual CaOx crystals in size, number, and localization. As clinically expected, they were more common in PHO than in SHO biopsies and were inconstant in KLTPHO samples. Other tested crystals (Ataza­navir, calcium phosphate) were not underlined by this fluorescence signal. There were no macrocrystals identified, nor >1 microcrystallite scoring in those patients (patient #24, #25, #26, #32, #35; see Tables S1 and S2).

Only few authors have reported ultrastructural observations of intracellular microcrystallites in the bone and in the kidney associated with oxalosis (Bacchetta *et al.*, 2015 ▸; Evan *et al.*, 2014 ▸; Saito *et al.*, 2016 ▸). Small punctiforms signals are not accessible to SEM because they are often intracellular. Their small size (1–10 µm) prevents reliable FTIRM characterization and probably explains why they have not been more thoroughly described (Worcester *et al.*, 2013 ▸). Like macrocrystals, these formations were more frequent and numerous in PHO patients than in SHO and KLTPHO patients, reinforcing the likeliness of signal specificity. Observation of microcrystallites in the kidney of control patients that had no known hyperoxaluria was not expected but could be of major interest, given the prevalence of CaOx kidney stone in the general population and the debate surrounding their formation (Bird & Khan, 2017 ▸).

A striking finding of our study is that synchrotron DUV fluorescence can detect diffuse intracellular signal. Like macro- and micro-crystallite density, the proportion of positive tubular sections and to a greater extent its quantification using randomly spotted tubular cell fluorescence measurement correlates with clinical severity (more frequent and lower in SHO than in PHO, KLTPHO, and control patients). It also correlated with the estimated glomerular filtration rate (eGFR) and urinary oxalate over creatinine ratio whereas there were no statistical links between these measurements and eGFR in control patients.

Given the low number of patients we cannot exclude confounding bias such as variations due to accumulation of other fluorescent compounds such as uric acid due to renal dysfunction. However, no tubular accumulation was spotted in stage 4 chronic kidney diseases (CKD4) control patients (patient 24, 25 and 31, respectively, graded 1, 1 and 0 in the nine-points histological score). One of the two tubular diffuse signal positive control biopsies is patient 34 in which subsequent analysis found a FTIR-confirmed CaOx microcrystal suggesting potential oxalate metabolism disturbance.

Altogether these elements strongly support that the observed fluorescence is indeed attributable to oxalate.

The ability to ‘see’ intracellular oxalate accumulation could lead to major progress in the understanding of the oxalate mediated kidney injury mechanism. In our experience, there is often a poor correlation between histological findings (few crystallites) and severe renal failure in renal oxalosis. Oxalate identification on a kidney allograft biopsy is associated with poorer renal survival (Palsson *et al.*, 2020 ▸; Pinheiro *et al.*, 2005 ▸; Bagnasco *et al.*, 2009 ▸). Recent studies suggest that high urinary oxalate excretion could be an independent risk factor of chronic kidney disease progression in the general population (Waikar *et al.*, 2019 ▸).

While it is established that oxalate calcium crystals are noxious for tubular epithelia (Thamilselvan & Khan, 1998 ▸), it has been postulated that oxalate itself, independently of its crystalline form, could be nephrotoxic, probably through triggering inflammation via interleukin-2 receptor (IL2R) signalling (Koul *et al.*, 2014 ▸; Thamilselvan *et al.*, 2014 ▸). Therefore, crystallization as a marker of local supersaturation could only reveal the tip of the iceberg of oxalosis-related pathology.

Tubular accumulation was often heterogeneous from one tubular section to another within the same biopsy. Determinants of this heterogeneity and correlation with markers of tubular injury would be of great interest.

Standard renal histology is not always sufficient to affirm the diagnosis of renal oxalosis. High urinary oxalate, genetic testing, enteric oxalate hyperabsorption enabling underlying condition and exclusion of another cause of tubulointerstitial disease are almost as important, if not more so, to formally establish a proper diagnosis (Lumlertgul *et al.*, 2018 ▸). Crystal density (0.25 crystals per glomeruli) has been proposed as a histological cut-off for renal oxalosis (Nasr *et al.*, 2008 ▸; Buysschaert *et al.*, 2020 ▸). We could not acquire large enough areas on all biopsies to determine such a ratio. Those elements were not considered in our sensitivity and specificity evaluation that likely overcomes standard polarized microscopy evaluation. However, synchrotron WFUV will probably never be used for routine renal oxalosis diagnosis but its ability to see beyond the macrocrystallized oxalate presence could be interesting in litigious cases.

Major progress has been made towards potential treatment of hyperoxaluric patients (Burns *et al.*, 2020 ▸; Estève *et al.*, 2019 ▸; Kletzmayr *et al.*, 2020 ▸; Le Dudal *et al.*, 2019 ▸; Liebow *et al.*, 2017 ▸). These new molecules call for solid follow-up markers and surrogate endpoint (Milliner *et al.*, 2020 ▸). Oxaluria interpretation in advanced kidney disease is challenging as the kidney’s ability to secrete oxalate dramatically decreases. Oxalemia can vary quickly in SHO patients. It is also highly dependent on renal excretion (and therefore GFR) which likely explains why its interest as a prognosis marker is still under debate (Hillebrand & Hoppe, 2020 ▸; Shah *et al.*, 2020 ▸). Oxalemia measurement can be challenging and is not well standardized (Stokes *et al.*, 2020 ▸). We believe that UV measurement of oxalate impregnation of the kidney could be a powerful tool to assess this new treatment efficiency especially after kidney transplantation since these patients often benefit from a close longitudinal follow-up with serial kidney sampling.

The main limitation of our study is the relatively low number of biopsies that we could analyse and incomplete clinical and biological information, due the fact that Tenon Hospital is a tertiary reference centre with late referral of patients. Since renal oxalosis is a rare disease, we could not test the diagnostic performance of our new technique in a naive population and could not determine positive and negative predictive values. A prospective larger study with better patient phenotyping is needed to support our observations.

Owing to its unique potential to identify crystallized and non-crystallized oxalate in kidney biopsies, synchrotron DUV fluorescence could not only help improve renal oxalosis diagnosis in litigious cases but open new paths of research towards the understanding of oxalate participation in kidney diseases and assess incoming hyperoxaluria treatment efficiency.

## Supplementary Material

Figure S1-S3 and table of patients with details. DOI: 10.1107/S1600577521011371/yn5078sup1.pdf

## Figures and Tables

**Table 1:** Contingency nine-points score showing sensitivity and specificity of the score

Nine-points oxalate histological score	PHO (*n* = 11)	SHO (*n* = 8)	KLTPHO (*n* = 7)	Control (*n* = 12)
Score (5–9)	9/11 (82%)	4/4 (50%)	4/3 (57%)	1/12 (8%)
*p* (Fischer exact test) versus control	0.0006	0.1089	0.0379	
Sensitivity/specificity	0.82 (0.52–0.97)	0.5 (0.22–0.78)	0.57 (0.25–0.84)	Sp 0.92 (0.64–1.00)
Score (6–9)	8/11 (73%)	4/4 (50%)	1/6 (14%)	0/12 (0%)
*p* (Fischer exact test) versus control	0.0003	0.0144	0.3684	
Sensitivity/specificity	0.73 (0.43–0.90)	0.5 (0.22–0.78)	0.14 (0.01–0.51)	Sp 1 (0.76–1.00)
