# Supplementary material for: Detection and localization of calcium oxalate in kidney using synchrotron deep ultraviolet fluorescence microscopy
Source: J Synchrotron Radiat. 2022 Jan 1;29(Pt 1):214–23. doi: 10.1107/S1600577521011371 (PMC8733991; doi:10.1107/S1600577521011371)
Supplement: Supplementary file 1 [file s-29-00214-sup1.pdf]

JOURNAL OF SYNCHROTRON RADIATION

Volume 29 (2022)

Supporting information for article:

Detection and localization of calcium oxalate in kidney using synchrotron deep ultraviolet fluorescence microscopy

Emmanuel Esteve, David Buob, Frederic Jamme, Chantal Jouanneau, Slavka Kascakova, Jean-Philippe Haymann, Emmanuel Letavernier, Louise Galmiche, Pierre Ronco, Michel Daudon, Dominique Bazin and Matthieu Réfrégiers

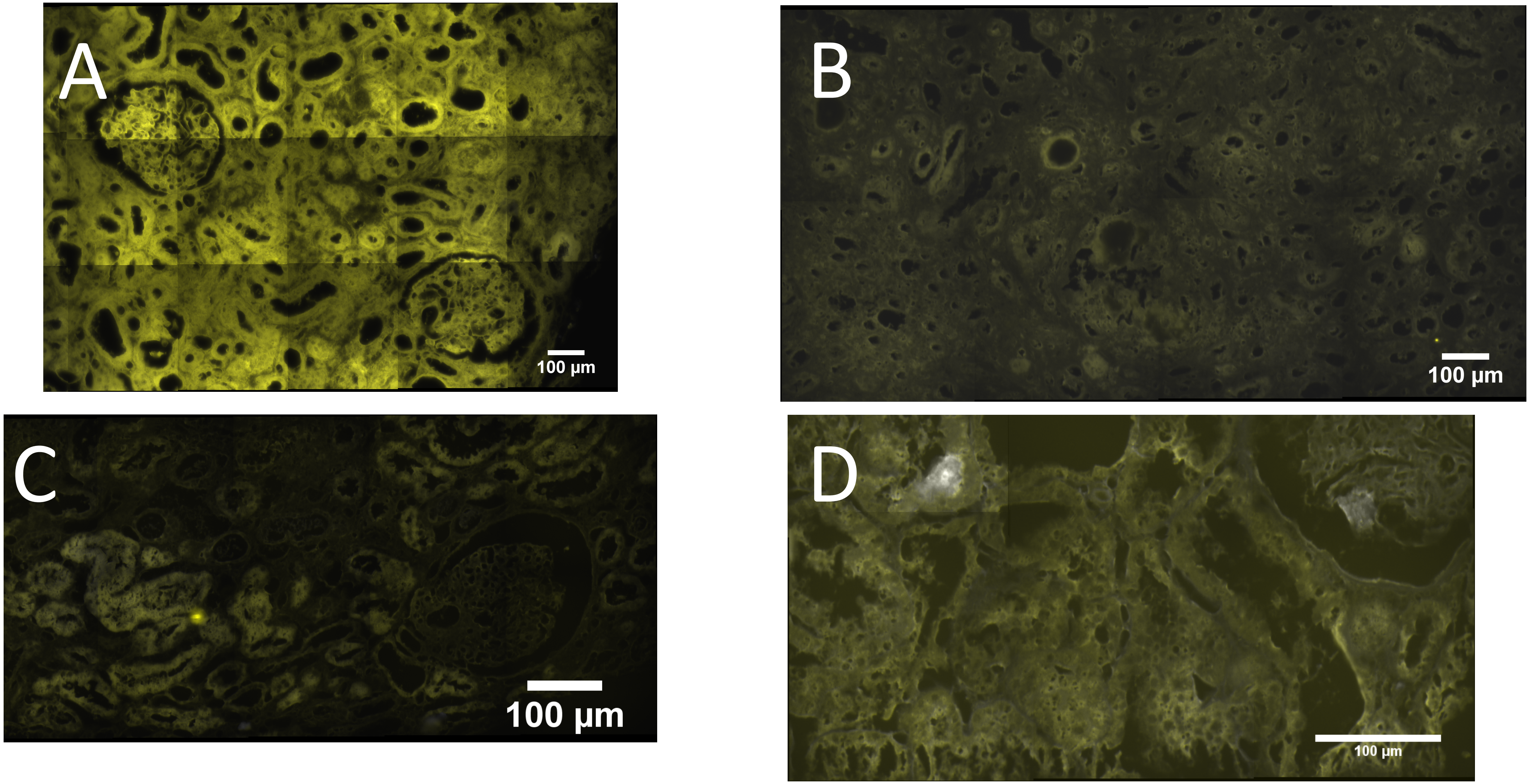

Supplementary Figure 1. WFUV mapping of the 4 excluded kidney biopsies. The signal (yellow) was obtained after substraction of the 412/438 nm channel and 327/353 nm channel fluorescence map. The background signal (grey) was obtained using the 327/353 nm channel (aromatic amino acid fluorescence). In 2 adenosinephosphoribosyltransferase deficient patients (A and B), in one suspected autosomal dominant tubulointerstitial kidney disease (C) and in one crystal-storing hystiocytosis nephropathy disease (D) signal was aberrantly positive over the whole studied tissue.

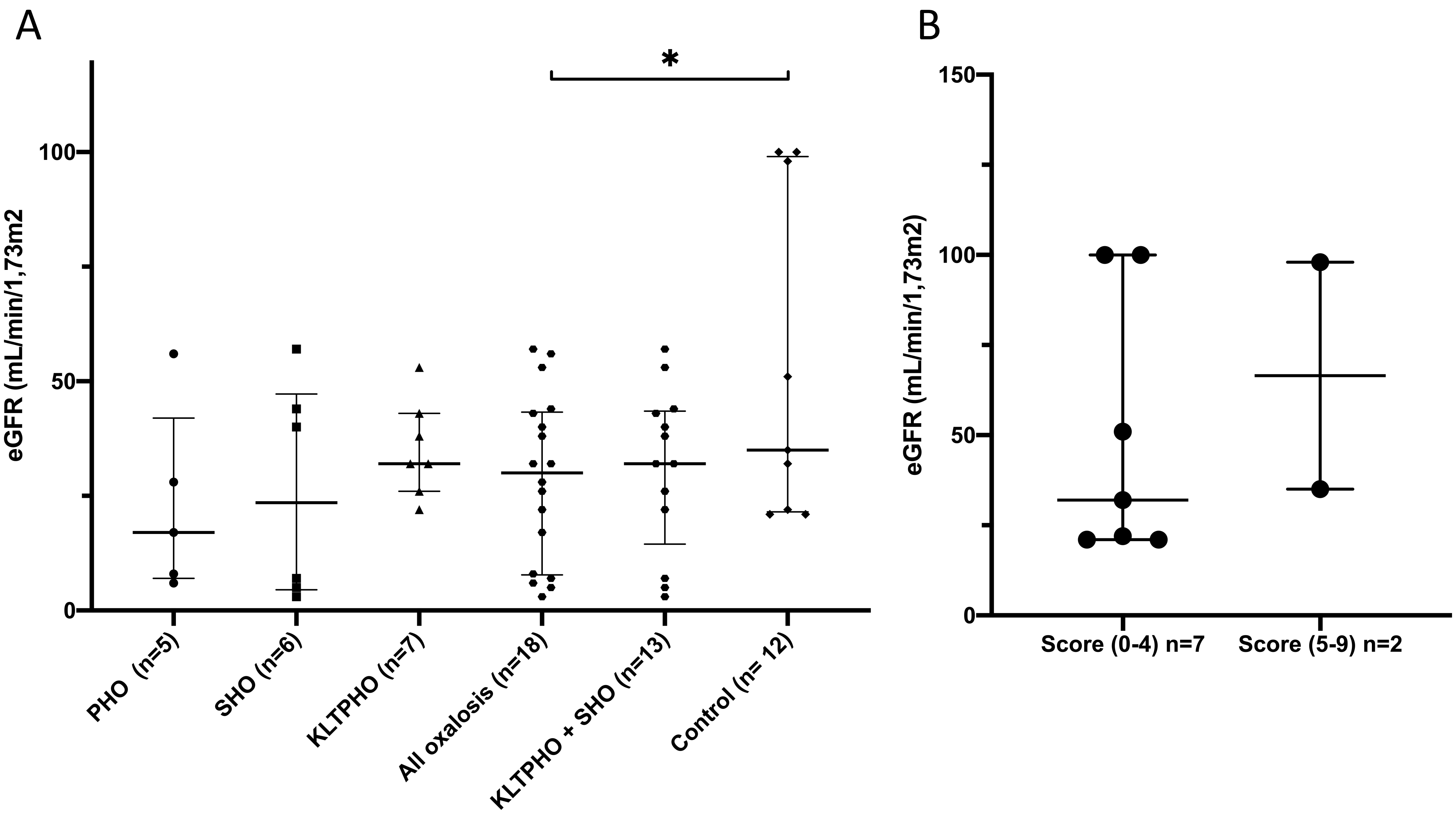

Supp. Figure 2. eGFR correlation in various subgroups. A. eGFR is widely distributed within the various subgroups but is significantly lower in oxalosis patients than in controls. B. In control patients the 9 points oxalosis histological score do not stratify patients according to their eGFR. Median and interquartile range 25-75 *:p<0,05.

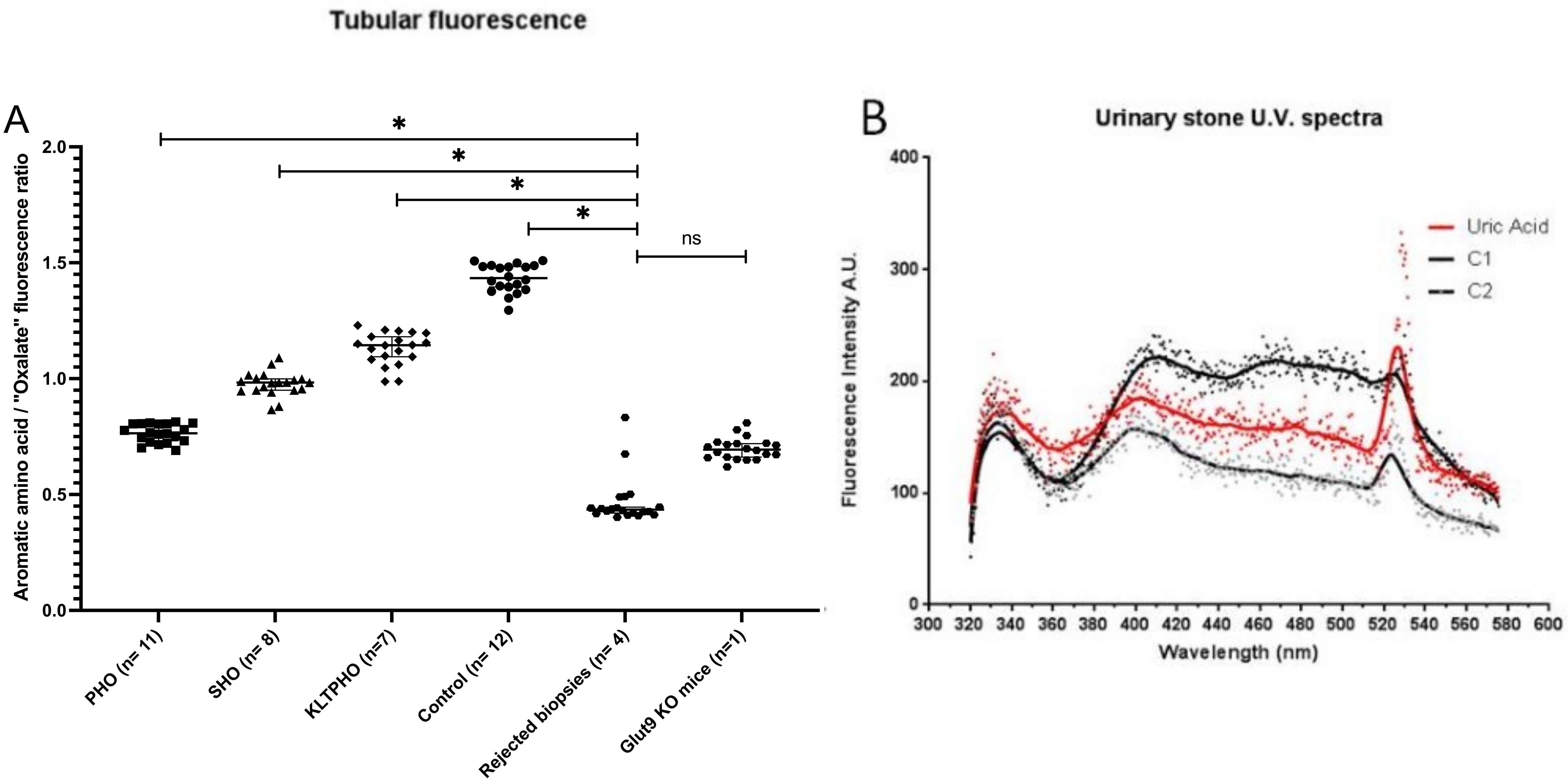

Supp. Figure 3. The 4 rejected biopsies (patients 36, 37, 38 and 39) show different fluorescent properties than oxalosis biopsies that could be related to purine metabolism derivative.
A. Tubular fluorescence ratio is significantly lower in these biopsies but in Glut9 knocked out mice.
Patient 36 has a clinical course suggestive of autosomal dominant tubulointerstitial kidney disease (ADTKD): ESRD in a first degree relative, juvenile gout with hyperuricemia.[33] Uromodulin mutation was not identified. As he was lost to follow-up, mutations of renin or less likely hnf1b or muc1 was not performed. However we can assume that he presented, as patient 37 (monoclonal light chain crystallization induced kidney disease), altered tubular proximal handling of uric acid.[34] Patient 38 and 39 suffered from DHA crystal nephropathy caused by adenine phosphoribosyltransferase deficiency. 2,8DHA and uric acid are two byproducts of purine metabolism that share common chemical backbone. It can be challenging to distinguish them using standard biochemical approaches.[18,35] Interestingly fluorescence properties of these 4 patients biopsies ressembles Glut9 knock out mice kidney that are known for their alteration of uric acid renal handling. *** : $p<0.001$. ns: non-significant.
B. UV emission spectra under 275nm excitation of uric acid (red), weddellite (C1, Calcium Oxalate Monohydrate) and whewellite (C2, Calcium Oxalate Dihydrate) kidney stones share common fluorescence properties around 420nm.

Patient	Sex	Age at biopsy	Clinical context	Histological diagnosis	Category	eGFR (ml/min/1,73m²)	oxaluria (mmol/mmol)	Microcystal. (0-3)	Macrocrystal. (0-1)	% of positive tub.	9 point score
1	*M*	*13*	*PHO type 1. AGT 10%. AKI 3 years post isolated kidney transplantation.*	*Renal oxalosis.*	*PHO*	*28*	*n.a.*	*3*	*1*	*10*	*7*
2	*F*	*25*	*PHO type 1. AGT 4%. 2 month post 2nd kidney 1st liver transplant systematic biopsy*	*Tubular suffering. Renal oxalosis.*	*KLTPHO*	*53*	*n.a.*	*2*	*1*	*0*	*5*
2	*F*	*31*	*PHO type 1. AGT 4%. 4 month post 3rd kidney 1st liver transplant sytematic biopsy*	*Interstitial fibrosis with tubular atrophy. Antibody mediated rejection. Few oxalate deposits*	*KLTPHO*	*43*	*0,11*	*1*	*0*	*5*	*2*
2	*F*	*21*	*PHO type 1. AGT 4%. 3 month post isolated 1st kidney transplant systematic biopsy*	*Normal kidney. Few oxalate deposits.*	*PHO*	*n.a.*	*0,20*	*2*	*1*	*95*	*8*
3	*M*	*36*	*PHO type 1 HTZ. AGT 41%. 1 Year systematic isolated renal 2nd transplant biopsy.*	*Mild interstitial fibrosis and tubular atrophy.*	*PHO*	*56*	*0,13*	*1*	*0*	*12,5*	*3*
4	*M*	*40*	*Post FTIRM analysis PHO diagnosis. CKD ESRD.*	*FTIRM confirmed renal oxalosis.*	*PHO*	*6*	*n.a.*	*0*	*0*	*85*	*3*
5	*F*	*33*	*CKD. PHO type 1, AGXT homozygous mutation.*	*FTIRM confirmed renal oxalosis. Interstitial fibrosis.*	*PHO*	*8*	*0,26*	*3*	*1*	*50*	*8*
6	*F*	*45*	*AKI 1 month after 1st isolated renal transplant.*	*FTIRM confirmed renal oxalosis.*	*PHO*	*17*	*n.a.*	*2*	*1*	*75*	*8*
7	*M*	*4 month*	*Nephrectomy. PHO.*	*FTIRM confirmed renal oxalosis.*	*PHO*	*ESRD*	*n.a.*	*0*	*1*	*40*	*5*
8	*M*	*n.a.*	*ESRD. Crystal. FTIRM led PHO type 1 diagnosis.*	*FTIRM confirmed renal oxalosis.*	*PHO*	*ESRD*	*n.a.*	*3*	*1*	*0*	*6*
9	*n.a.*	*n.a.*	*Nephrectomy. PHO.*	*FTIRM confirmed renal oxalosis.*	*PHO*	*ESRD*	*n.a.*	*3*	*1*	*0*	*6*
10	*n.a.*	*n.a.*	*Nephrectomy. PHO.*	*FTIRM confirmed renal oxalosis.*	*PHO*	*ESRD*	*n.a.*	*3*	*1*	*90*	*9*
11	*n.a.*	*n.a.*	*Nephrectomy. PHO.*	*FTIRM confirmed renal oxalosis.*	*PHO*	*ESRD*	*n.a.*	*3*	*1*	*55*	*9*
12	*M*	*50*	*Acute kidney injury. Ethylen glycol poisoning*	*Acute tubular necrosis. FTIRM confrimed renal oxalosis.*	*SHO*	*5*	*0,25*	*3*	*1*	*70*	*9*
13	*F*	*55*	*Acute kidney injury. Gastric bypass.*	*Acute tubular necrosis. FTIRM confirmed renal oxalosis.*	*SHO*	*44*	*n.a.*	*1*	*1*	*0*	*4*
14	*M*	*69*	*Chronic kidney disease. Short bowel disease due to mesenteric ischemia due to primary polycithemia.*	*Tubulo interstitial atrophic fibrosis. Renal oxalosis.*	*SHO*	*40*	*0,12*	***1***	*0*	*15*	*3*
15	*M*	*87*	*AKI/CKD. Diabetes, calcifying pancreatitis, dehydratation.*	*FTIRM confirmed renal oxalosis*	*SHO*	*3*	*0,13*	*0*	*1*	*75*	*6*
16	*M*	*61*	*Transient AKI. Cirrhosis. Gastric bypass.*	*FTIMR confirmed renal oxalosis. Acute tubular necrosis.*	*SHO*	*7*	*n.a.*	*3*	*1*	*0*	*6*
17	*M*	*34*	*Cystic fibrosis. IgA nephropathy. Nephrotic range proteinuria 10 years post 1st kidney transplant.*	*Chronic fibrosis. IgA glomerulonephritis recurrence. FTIRM confirmed oxalate deposits.*	*SHO*	*57*	*n.a.*	*1*	*1*	*0*	*4*
18	*M*	*65*	*Acute kidney injury. Gastrectomy for cancer.*	*FTIRM confirmed renal oxalosis.*	*SHO*	*na*	*na*	*3*	*1*	*62,5*	*9*
19	*M*	*69*	*Chronic kidney disease. Diabetes. Chronic pancreatitis.*	*FTIRM confirmed renal oxalosis.*	*SHO*	*na*	*na*	*3*	*0*	*5*	*4*
20	*M*	*16*	*PHO type 1. AGT 10%. AKI 2,5 years post KLTPHO*	*Mild renal oxalosis.*	*KLTPHO*	*32*	*n.a.*	*2*	*0*	*100*	*5*
21	*F*	*16*	*PHO. CKD 11 years post KLTPHO. Proteinuria.*	*Chronic and acute humoral transplant rejection. Acute tubular necrosis. No oxalate deposit (PM).*	*KLTPHO*	*32*	*n.a.*	*0*	*0*	*0*	*0*
22	*M*	*12*	*PHO type 1. 11 years post KLTPHO. CKD*	*Moderate interstitial fibrosis with tubular atrophy. CNI toxicity.*	*KLTPHO*	*38*	*n.a.*	*0*	*0*	*0*	*0*
23	*F*	*57*	*KLTPHO (Double HTZ AGXT mutation) M1. AKI.*	*FTIRM confirmed oxalosis recurrence. Acute humoral transplant rejection.*	*KLTPHO*	*26*	*0,25*	*3*	*1*	*50*	*8*
23	*F*	*57*	*KLTPHO (Double HTZ AGXT mutation) M6. AKI.*	*FTIRM confirmed oxalosis recurrence. Acute tubular necrosis.*	*KLTPHO*	*22*	*0,28*	*2*	*0*	*75*	*5*
24	*F*	*76*	*Chronic kidney disease. HIV.*	*FTIRM confirmed atazanavir crystal nephropathy.*	*Ctrl*	*21*	*n.a.*	*1*	*0*	*0*	*1*
25	*M*	*57*	*Acute kidney injury, hypercalcemia, chronic kidney disease, sarcoidosis, HIV.*	*Renal sarcoïdosis.*	*Ctrl*	*22*	*n.a.*	*1*	*0*	*0*	*1*
26	*M*	*64*	*Acute kidney injury, hypercalcemia, sarcoïdosis.*	*Renal sarcoïdosis. Interstitial calcifications.*	*Ctrl*	*51*	*n.a.*	*1*	*0*	*0*	*1*
27	*F*	*49*	*Isolated hematuria. Basalopathy.*	*Normal kidney. No collagen IV expression alteration.*	*Ctrl*	*98*	*n.a.*	*1*	*1*	*0*	*4*
28	*M*	*57*	*Preimplantatory kidney transplant biopsy.*	*Normal kidney. Mild tubular suffering.*	*Ctrl*	*100*	*n.a.*	*0*	*0*	*0*	*0*
29	*F*	*55*	*Preimplantatory kidney transplant biopsy.*	*Normal kidney*	*Ctrl*	*100*	*n.a.*	*0*	*0*	*0*	*0*
30	*F*	*62*	*Chronic kidney disease. Proteinuria. VEGF inhibitor exposition.*	*Glomerular thrombotic microangiopathy.*	*Ctrl*	*32*	*n.a.*	*0*	*0*	*0*	*0*
31	*F*	*32*	*Postpartum haemorrhage acute kidney injury.*	*Glomerular thrombotic microangiopathy and acute tubular necrosis.*	*Ctrl*	*21*	*n.a.*	*0*	*0*	*0*	*0*
32	*F*	*57*	*Post transplant systematic renal biopsy.*	*FTIRM confimed amorphous carbonated calcium phosphate deposits.*	*Ctrl*	*n.a.*	*n.a.*	*0*	*0*	*0*	*0*
33	*n.a.*	*n.a.*	*Post transplant systematic renal biopsy.*	*Normal Kidney*	*Ctrl*	*n.a.*	*n.a.*	*0*	*0*	*10*	*1*
34	*M*	*7*	*AKI 2 month after first kidey transplantation for nephrotic syndrom.*	*Acute cellular rejection. Few subsequent FTIRM confirmed oxalate deposits.*	*Ctrl*	*35*	*n.a.*	*0*	*1*	*22,5*	*5*
35	*F*	*75*	*Nephrangiosclerosis. Nephrocalcinosis. Sjogren disease.*	*FTIRM confimed amorphous carbonated calcium phosphate deposits.*	*Ctrl*	*n.a.*	*n.a.*	*1*	*0*	*0*	*1*
36	*M*	*24*	*Chronic kidney disease. Juvenile gout and hyperuricemia.*	*Chronic tubulointerstitial nephritis.*	*EXCLUDED*	*37*	*n.a.*	*1*	*0*	*75*	*4*
37	*M*	*69*	*Monoclonal light chain myeloma.*	*Crystal storing hystiocytosis. Proximal tubular alterations.*	*EXCLUDED*	*55*	*n.a.*	*2*	*1*	*80*	*8*
38	*F*	*72*	*CKD.*	*FTIRM confirmed DHA.*	*EXCLUDED*	*n.a.*	*n.a.*	*1*	*0*	*75*	*4*
39	*F*	*61*	*3 month post renal transplantation renal dysfunction.*	*FTIRM confirmed DHA.*	*EXCLUDED*	*n.a.*	*n.a.*	*1*	*0*	*50*	*3*
